# Supplementary figures and images for: Combined Inhibition of TGF-β Signaling and the PD-L1 Immune Checkpoint Is Differentially Effective in Tumor Models
Source: Cells. 2019 Apr 5;8(4):320. doi: 10.3390/cells8040320 (PMC6523576; doi:10.3390/cells8040320)

Figure S1

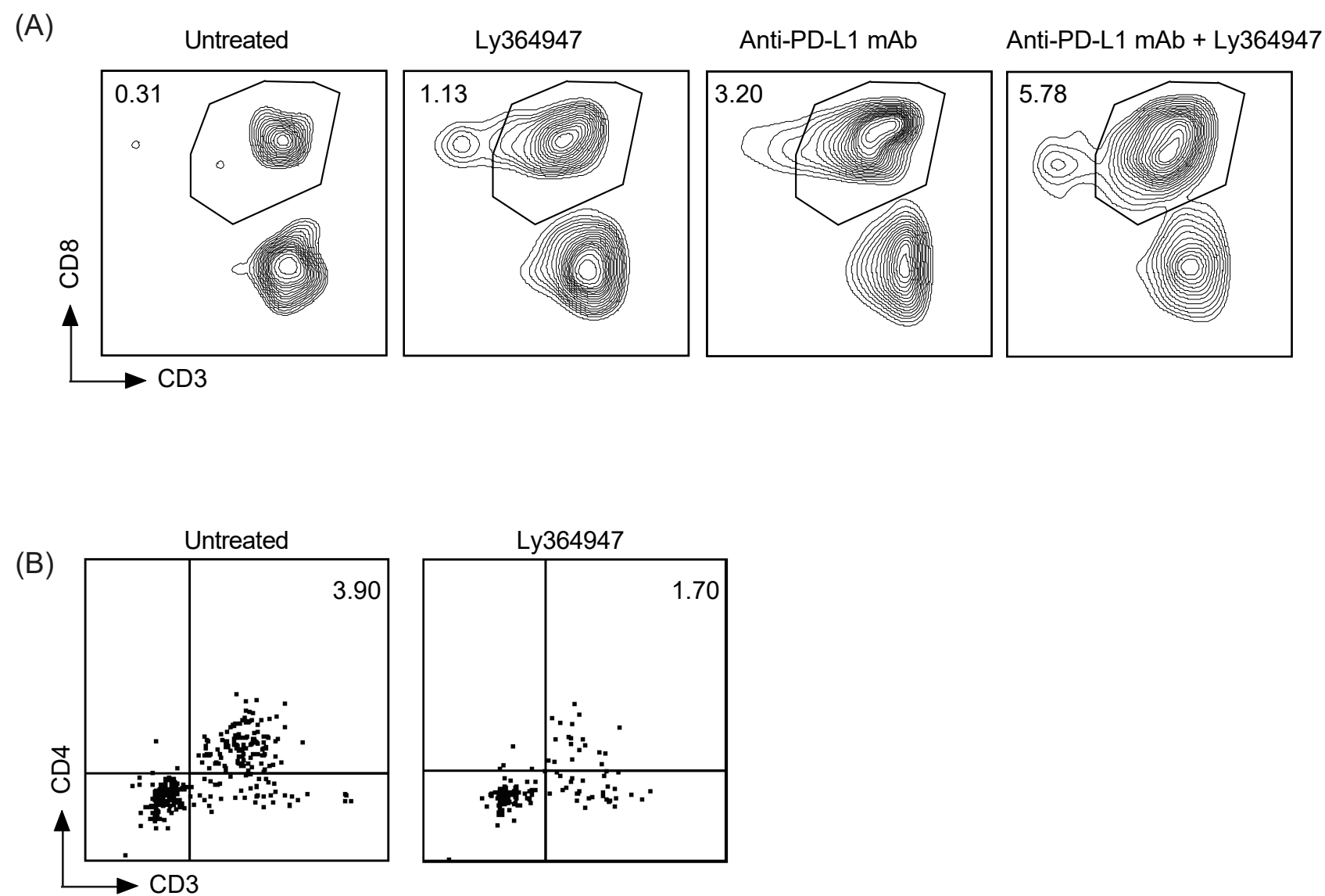

Figure S2

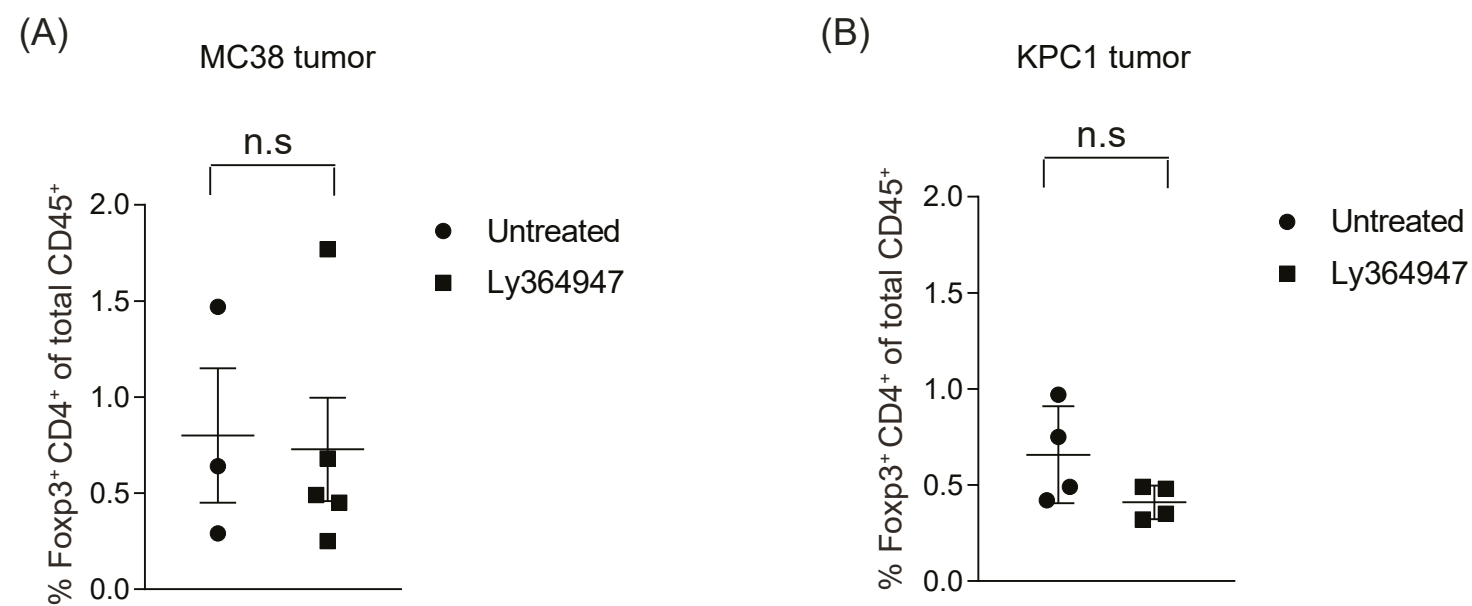

Supplement: Supplementary file 1 [file cells-08-00320-s001.pdf]
